# Oral Administration of Bacterial *β* Cell Expansion Factor A (BefA) Alleviates Diabetes in Mice with Type 1 and Type 2 Diabetes

**DOI:** 10.1155/2022/9206039

**Published:** 2022-02-10

**Authors:** Huan Wang, Jing Wei, Hong Hu, Fuyin Le, Heng Wu, Hong Wei, Jie Luo, Tingtao Chen

**Affiliations:** ^1^National Engineering Research Center for Bioengineering Drugs and the Technologies, Institute of Translational Medicine, Nanchang University, Nanchang 330031, China; ^2^Precision Medicine Institute, The First Affiliated Hospital, Sun Yat-sen University, Guangzhou 510080, China; ^3^School of Public Health and Key Laboratory of Preventive Medicine, Nanchang University, Nanchang 330031, China

## Abstract

Diabetes mellitus (DM) is a group of metabolic diseases, and there is an urgent need to develop new therapeutic DM oral drugs with fewer side effects and sound therapeutic efficacy. In this study, a *β* cell expansion factor A (BefA) production strain of *Escherichia coli* (BL21-pet 28C-BefA) was constructed, and the antidiabetes effect of BefA was evaluated using type 1 DM (T1DM) and type 2 DM (T2DM) mice models. The T1DM mice results indicated that BefA significantly reduced blood glucose levels; exerted a protective effect on islet *β* cell morphology; downregulated the expressions of TLR-4, p-NF*κ*B/NF*κ*B, and Bax/Bcl-2, and the secretion levels of IL-1*β* and TNF-*α*; increased the expression of PDX-1 protein and insulin secretion in a concentration-dependent manner; and restored the disturbed microbial diversity to normal levels. Similarly with the T1DM mice, BefA obviously increased islet *β* cells and reduced the inflammatory reaction and apoptosis in T2DM mice, as well as improved liver lipid metabolism by downregulating the expressions of CEBP-*α*, ACC, and Fasn; inhibited the synthesis of triglycerides; and induced Cpt-1, Hmgcs2, and Ppar*α* in a concentration-dependent manner. In conclusion, BefA alleviates diabetes via increasing the number of islet *β* cells, reducing the inflammatory reaction and apoptosis, improving liver lipid metabolism, and restoring microbial diversity to normal levels, which provides a new strategy for a DM oral drug.

## 1. Introduction

Diabetes mellitus (DM) is a group of complex metabolic disorders characterized by abnormally elevated blood glucose concentrations secondary to either insufficient insulin secretion, insulin resistance, or both [1, 2].Studies show that acute complications (e.g., hyperosmolar coma and diabetic ketoacidosis) can be caused by elevated blood glucose, which eventually cause damage to the liver, heart, cerebrovascular, and other organs and even lead to death [3, 4].The global mortality rate of DM is as high as 10.7%, and it is estimated that there will be 693 million people with DM worldwide by 2045 [5]. The increasing prevalence of DM and its high medical expenses makes it an urgent public health problem all over the world [6, 7].

Insulin-dependent type 1 diabetes mellitus (T1DM) and insulin-independent type 2 diabetes mellitus (T2DM) are the main types of diabetes, among which T1DM results from the specific deficiency of insulin-producing pancreatic *β* cells from autoimmune destruction [8] and T2DM is an age-related disease characterized by the dysfunction of glucose metabolism representing insulin-resistant states that is accompanied by a destruction of *β* cells [9]. For the treatment of T1DM, insulin injection therapy was applied after its discovery in 1922, and it can only alleviate (but fails to eliminate)T1DM and also may cause long-term physical suffering through subcutaneous injections [10]. New methods of therapy, such as immunotherapy, gene therapy, and organ transplantation, have been developed rapidly, but they are still in the research stage due to problems, such as therapeutic side effects, safety issues, or insufficient donors [11–13]. For the treatment of T2DM, various drugs have been developed, but people have found their defects during clinical practice. For example, biguanide drugs, such as metformin, can lead to macrocytic anemia and increase the burdens of the liver and kidneys [14]; sulfonylurea drugs can enhance insulin sensitivity but can cause digestive system disease and impairment of liver function [15], and new drugs, such as GLP-1 receptor agonists, have a remarkable curative effect but their expensive cost and the need for injection limit their clinical use [16]. Based on the disadvantages of the above treatment strategies, it is of great importance to develop new therapeutic drugs for both T1DM and T2DM with fewer side effects and better therapeutic efficacy. Both impaired pancreatic *β* cell function and insulin secretion have been demonstrated in both T1DM and T2DM,while treatments targeting pancreatic *β* cell proliferation are currently lacking [17, 18].

Many studies have proven that intestinal microbes and their metabolites exert important effects on obesity and blood glucose metabolism, but the direct evidence is not clear. In 2016, a research article published in *eLife* reported an intestinal microbiota-derived protein named *β* cell expansion factor A (BefA) that could induce pancreatic *β* cell proliferation in the early development of zebrafish. More meaningfully, the research team discovered that the BefA protein homologues in human intestinal microbial metabolites share the same proliferative effect. Besides, since the BefA protein is derived from intestinal microbes, it possesses high tolerance to the intestinal environment when compared to other drugs and can be administered orally to avoid the physiological pain to patients caused by repeated injections, indicating that the BefA protein may be a new strategy for treatment of DM [19].

The pancreatic islet *β* cells of T1DM mice are irreversibly destroyed by abnormal autoimmune attack under normal pathological conditions [20]. Some key proteins are associated with apoptosis, such as pancreas/duodenum homeobox protein 1 (PDX-1), which is a marker of islet *β* cell differentiation, maturation- and proliferation [21, 22]. Similarly, v-maf musculoaponeurotic fibrosarcoma oncogene homolog A (Mafa) and neurogenin-3 are critical transcription regulators in inducing *β* cell development and regeneration [23]. Furthermore, chronic systemic inflammation plays a promoting role in the occurrence and development of T2DM [24]; the key inflammatory proteins include TLR-4, p-NF*κ*B/NF*κ*B, IL-1*β*, and TNF-*α*.

Based on the irreversible damage of pancreatic *β* cells in T1DM and T2DM, this work shed light on the role of the increasing effect of the BefA protein by adjusting blood glucose level, body weight, and gene expression changes associated with *β* cell proliferation and necrosis. In addition, as disordered intestinal microbiota can lead to autoimmune damage in islet *β* cells and induce T1DM [25] and T2DM is prone to occur in obese individuals, with a high-fat diet possibly inducing fatty liver and liver inflammation [26], we also evaluated the effects of the oral administration of BefA on intestinal microbiota and liver function. In the present study, we constructed the BefA yield strain, isolated and purified the BefA protein, and evaluated its therapeutic effect and potential mechanisms in T1DM and T2DM mice for the first time, providing basic data for its clinical application.

## 2. Materials and Methods

### 2.1. Construction of the BefA Yield Strain and Protein Purification

The BefA gene (M001_10165) was codon-optimized for expression in *Escherichia coli* BL21 to favor higher protein yield and was synthesized with a histidine (His) tag (to facilitate the identification and purification of the BefA protein), which was inserted into the prokaryotic expression vector pet 28CfromKingsy Biotechnology Co. (Nangjing, China) to form the recombinant plasmid pet 28C-BefA. Then, the pet 28C-BefA was transformed into the *E.coli* BL21 strain to generate the BefA production strain of BL21-pet 28C-BefA.

To produce the BefA protein, the BL21-pet 28C-BefA strain was cultivated in Luria-Bertani (LB) medium (Solarbio Life Sciences, China, L1010) with kanamycin (50 *μ*g/ml; Solarbio Life Sciences, China, K1030) at 37°C. When the optical density value reached 0.6-0.8, 1 mM of isopropyl *β*-D-thiogalactoside (IPTG, Solarbio Life Sciences, China, I8070) was added into the culture medium to stimulate massive protein expression during the following 6 h cultivation. Then, the culture medium was centrifuged at 8,000 g for 30 min to obtain the bacterial pellet, which was further used for ultrasonic disintegration to flow out bacterial proteins. The BefA protein was purified with His-tag nickel beads (7Sea Biotech, China, PAN001-001C), and the purity and accuracy of the BefA protein were detected by SDS-PAGE electrophoresis and Western blotting. The purified protein concentration was determined by the BCA protein assay kit (Thermo Fisher Scientific, USA, 23227) according to the manufacturer's guidelines.

### 2.2. Construction and Intervention of DM Mice

To check whether BefA can affect the proliferation of *β* cells, newborn germ-free mice (GF group; *n* = 3), newborn SPF mice (SPF group; *n* = 3), and newborn germ-free mice treated with 1 ng BefA/g body weight (GFB group; *n* = 3) were used. For germ-free mice, after the birth of mice born by female germ-free mice in aseptic isolation, the sterilized mice were transferred into aseptic isolation via an isolation bin; BefA was given via oral gavage by skilled test staff.

For the T1DM mice model, 8-week male wild-type C57BL/6 mice (purchased from SJA Laboratory Animal Co., Ltd., China) were housed in specific pathogen-free conditions with an optimum environment (12 h light/dark cycle with ad libitum access to standard laboratory chow and water, humidity 50 ± 15%, temperature 22 ± 2°C). After acclimating for 1 week, some measures meant to avoid the effects of cages on the microbiome were carried out, including separating the mice individually in isolation bins, ensuring the same diet and sterile padding, and changing gloves frequently when changing the cages. The mice were injected intraperitoneally with streptozotocin (STZ, 50 mg/kg/d) for 5 consecutive days until the blood glucose concentration rose to 11.1 mM (199.8 mg/dl) or above and stabilized for 7 days, and then the mice were divided into three groups: (1) M group: T1DM model group treated with 0.9% physiological saline containing 0.01% gelatine administered intragastrically every other day for 14 times (*n* = 15); (2) MB10 group: T1DM model group treated with 0.9% physiological saline containing 0.01% gelatine and 10 *μ*g BefA administered intragastrically every other day for 14 times (*n* = 15); and (3) MB50 group: T1DM model group treated with 0.9% physiological saline containing 0.01% gelatine and 50 *μ*gBefA administered intragastrically every other day for 14 times (*n* = 15). Another 15 wild-type C57BL/6 mice were used as the normal control group (C group).Within each group, all mice were used to test blood glucose level (once a week), body weight (once a week), and fecal microbiota structure (at week 7), in which four mice were sacrificed for pancreas Western blotting analysis, four mice were sacrificed for pancreas qPCR analysis, and three mice were sacrificed for pancreas hematoxylin and eosin (HE) staining, immunohistochemical staining, and immunofluorescent staining.

For the T2DM mice model,8-week-old male wild-type C57BL/6 mice were acclimated for 1 week, then fed a high-fat diet (Research Diets, USA, D12492) for 6 weeks combined with intraperitoneal injection of a low concentration STZ (30 mg/kg) until the blood glucose concentration rose to 11.1 mM (199.8 mg/dl) or above and stabilized for 7 days [27–29]. Then, the mice were divided into five groups with15 mice each: (1) C group: mice fed with laboratory chow diet as the normal control group; (2) M group: mice treated with 0.9% physiological saline containing 0.01% gelatine administered intragastrically every other day for 14 times (*n* = 15); (3) MB5 group: mice treated with 0.9% physiological saline containing 0.01% gelatine and 5 *μ*gBefA (*n* = 15); (4) MB20 group: mice treated with 0.9% physiological saline containing 0.01% gelatine and 20 *μ*gBefA (*n* = 15); and (5) MB40 group: mice treated with 0.9% physiological saline containing 0.01% gelatine and 40 *μ*gBefA (n =15). Within each group, all mice were used to test blood glucose level (once a week), body weight (once a week), and glucose tolerance (at week 10), in which four mice were sacrificed for pancreas and liver Western blotting analyses, four mice were sacrificed for pancreas and liver qPCR analyses, three mice were sacrificed for pancreas immunohistochemical staining and liver oil red staining, and three mice were sacrificed for pancreas immunofluorescent staining. The BefA concentrations used in the T1DM and T2DM mice models were determined preexperimentally based on the concentration previously used for zebrafish [19].

### 2.3. Glucose Tolerance Test for DM Mice

Mice were fasted for 12 h prior to the test. Glucose (1.5 mg/g) was injected intraperitoneally, and blood glucose levels were measured at 0, 30, 60, 90, and 120 min after the injection.

### 2.4. Pathological Histology

Pancreas sections were fixed in 4% paraformaldehyde, embedded in paraffin, cut into 5 *μ*m sections, and rehydrated by xylene and declining grades of ethanol for 5 min. Then, they were washed three times for HE staining. Immunohistochemical and immunofluorescent tests were performed using anti-PDX-1 (Abcam, UK, ab47383) antibody and anti-insulin (Cell Signaling Technology, USA, #4590S) antibody. Frozen livers were sliced, and Oil Red O staining was performed as described earlier [30, 31] using the Oil Red O Stain Kit (Lipid Stain) (SenBeiJia Biological Technology, China, BP-DL101). According to the instructions of the kit, the Oil Red O solution was added dropwise onto the tissue for a 5-10 min incubation period. Excess staining buffer was removed with 85% propylene glycol. The tissues were washed with distilled water and counterstained with hematoxylin.

### 2.5. Western Blotting Analysis

Tissues were lysed in radioimmunoprecipitation assay (RIPA) lysis buffer (Solarbio Life Sciences, China, R0010) and centrifuged at 8,000 g for 15 min at 4°C after sonication on ice. The supernatants were collected, and the protein concentrations were measured by BCA assay. Equal amounts of sample (60 mg/lane) were heat denatured in loading buffer and separated via 10% gel electrophoresis (SDS-PAGE) and were transferred onto a PVDF membrane (Millipore, Germany, IPVH00010). Then, the membrane was blocked with 5% skim milk-TBST solution (20 mM Tris-HCl (pH 7.6), 127 mMNaCl, and 0.1% Tween 20) for 1 h at room temperature [32]. After being washed three times with TBST, the samples were incubated overnight with primary antibodies directed against anti-*β*-actin (ABclonal, USA, AC026), anti-His tag (Solarbio Life Sciences, China, K200060M), anti-toll-like receptor-4 (TLR-4) (Santa Cruz Biotechnology, USA, sc-293072), anti-nuclear factor kappa-B (NF*κ*B) (Abcam, UK, ab32360), anti-phosphorylated nuclear factor kappa-B (p-NF*κ*B) (Santa Cruz Biotechnology, USA, sc-101751), anti-Bcl-2-associated X protein (Bax) (Cell Signaling Technology, USA,# 5023S), anti-B cell lymphoma-2 (Bcl-2) (Cell Signaling Technology, USA,#3498S), and anti-CCAAT enhancer binding protein-*α* (CEBP-*α*) (Cell Signaling Technology, USA,#2295S) at 4°C. The membranes were then washed three times with TBST and incubated with horseradish peroxidase- (HRP-) linked anti-rabbit IgG or anti-mouse IgG antibodies at room temperature for 1 h. Membrane-bound immune complexes were detected by an enhanced chemiluminescence system (Thermo Scientific, USA). Quantification was performed by densitometric analysis using ImageJ software (NIH). All Western blotting experiments of each protein were carried out with four experimental replications (see in supplementary Figure S1; the legend was described at the end of this manuscript).

### 2.6. RNA Extraction and qPCR

Mouse pancreas and liver were homogenized in TRIzol Reagent (Life Technologies, USA, 15596026) prior to RNA extraction, and PCR primers were designed using Primer 5.0. qPCR and amplification were performed by the ABI 7900HT fast real-time PCR system (Applied Biosystems, USA). The reaction mixture contained 10 *μ*l of SYBR® Primer EX Taq II (Takara, Japan, RR420A), 0.4 *μ*l ROX reference dye (50×) (Takara, Japan, RR420A), 1.0 *μ*l DNA template, and 0.8 *μ*l of each of the primers (final concentration was 0.4 *μ*M), with 7 *μ*l Milli-Q H_2_O. The qPCR condition was as follows: start at 95°C for 10 min, followed by 40 cycles of degeneration at 95°C for 30 s, annealing at 60°C for 30 s, and extension at 72°C for 30 s. Relative levels (fold change) of the target genes were normalized against a housekeeping gene (GAPDH) and analyzed by the 2^−(△△Ct)^ method (for specific primers, see Table 1).

### 2.7. High-Throughput Sequencing Analyses

The feces of mice were collected and were stored at -80°C. Bacterial genomic DNA from the feces was obtained using a DNA extraction kit (Tiangen, China, DP302). DNA samples were amplified targeting the V3-V4 region of the bacterial 16S rRNA gene using 338F/806R primers [33]. Bioinformatic analysis was performed with UPARSE software version 7.0.100 (http://drive5.com/uparse/) using the UPARSE operational taxonomic units (OTUs). Sequences with ≥97% similarity were assigned to the same OTUs. Weighted UniFrac distance analysis was performed using the quantitative insights into microbial ecology (QIIME) software package version 1.9.1 (http://qiime.org/; QIIME Development Team), and the linear discriminant analysis effect size (LEfSe) method was used to analyze the bacteria with significant differences among the C, M, MB10, and MB50 groups (GenBank accession no. PRJNA637680) [34].

### 2.8. Statistical Analysis

Data handling, analyses, and graphical representations were performed using GraphPad Prism version 7.0 (GraphPad Software, Inc. USA). Values are shown as mean ± SD. Statistical significance was determined using one-way or two-way ANOVA and was annotated using the international convention related to the statistical representation.

## 3. Results

### 3.1. Increasing Effect of Purified BefA on the Number of Islet *β* Cells in Newborn Mice

**Figure 1:**
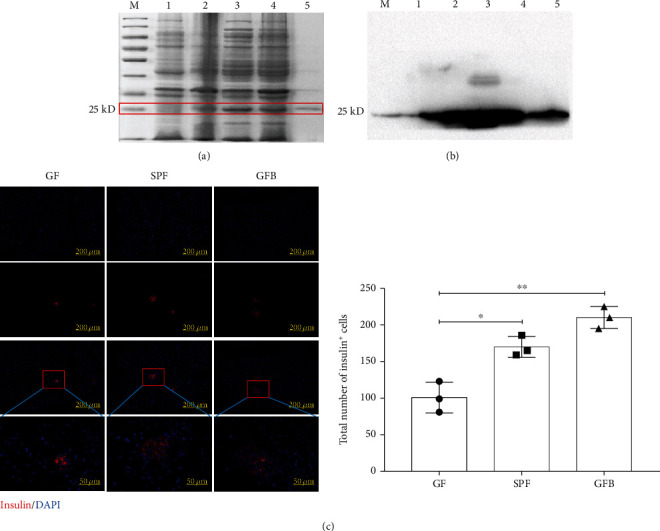
Increasing effect of purified BefA on the number of islet *β* cells in newborn mice. (a) Protein purity detection using SDS-PAGE. (b) Verifying the band position of the BefA protein using Western blotting. (c) BefA increased the number of islet *β* cells in GF mouse. The red area marks secreted insulin, which is used to label islet *β* cells. M: protein marker. (1) Bacteria after IPTG induction. (2) Bacterial lysate supernatant. (3) Bacterial lysate precipitation. (4) Supernatant purified protein. GF: germ-free mouse (*n* = 3); SPF: specific pathogen-free mouse (*n* = 3); GFB: germ-free mouse + BefA protein treatment (*n* = 3, 1 ng BefA/g body weight).

To obtain the purified BefA protein, the codon optimized BefA gene was inserted into the prokaryotic expression vector pet 28C to make the BefA production strain of BL21-pet 28C-BefA. As shown in Figure 1(a), the massive amount of BefA produced by the BL21-pet 28C-BefA strain was presented as a soluble protein, and the purity of soluble BefA protein reached above 98%.Their accuracy (25-28 kD in size) was further confirmed using Western blotting results (Figure 1(b)).

As no work was done to verify the increasing effect of orally administered BefA on the number of mammalian islet *β* cells, newborn germ-free (GF) mice and specific pathogen-free (SPF) mice were used in the present study to confirm the antidiabetes effect of BefA for the first time, and our results indicated that BefA could markedly increase the number of *β* cells compared among the SPF mice and GF mice (Figure 1(c)), which is consistent with previous work in zebrafish [19].

### 3.2. BefA Protein Significantly Reduced Pancreatic Inflammation in T1DM Mice

**Figure 2:**
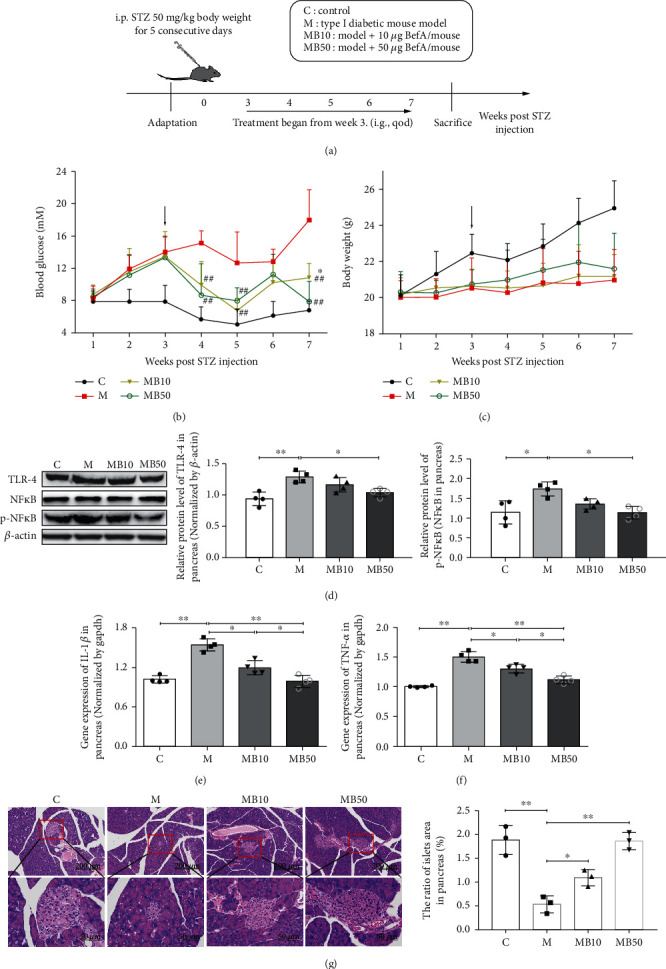
BefA protein significantly reduced pancreatic inflammation in T1DM mice. (a) Scheme of animal experiment. (b) Blood glucose levels tested from mice tail veins. (c) Body weight. Western blotting results of the expression level of proinflammatory proteins, including TLR-4 and the ratio of p-NF*κ*B/NF*κ*B in the pancreas of T1DM mice (d), gene expression levels of IL-1*β* (e), and TNF-*α* (F) in the pancreas measured by qPCR. (g) Results of H&E staining (×100 and ×400). The black arrows in (a) and (b) indicate that the administration of BefA started at week 3. C group: wild-type C57BL/6 mice (*n* = 15); M group: T1DM model group treated with 0.9% physiological saline containing 0.01% gelatine administered intragastrically every other day for 14 times (*n* = 15); MB10 group: T1DM model group treated with 0.9% physiological saline containing 0.01% gelatine and 10 *μ*g BefA administered intragastrically every other day for 14 times (*n* = 15); and MB50 group: T1DM model group treated with 0.9% physiological saline containing 0.01% gelatine and 50 *μ*g BefA administered intragastrically every other day for 14 times (*n* = 15). Data are presented as mean ± SD. From (b, c), ^∗^ means significant difference compared with the C group, *p* < 0.05; ## means significant difference compared with the M group, *p* < 0.01. From (d)–(f), ^∗^*p* < 0.05 and ^∗∗^*p* < 0.01.

To evaluate the therapeutic effect of BefA in the mammalian model for DM, we established a T1DM mice model divided into four groups, including the C group (normal control group), M group (T1DM model group), MB10 group, and MB50 group (Figure 2(a)). As shown in Figure 2(b), 10 *μ*g BefA and 50 *μ*g BefA could significantly reduce the blood glucose levels in a concentration-dependent manner (*p* < 0.05) (Figure 2(b)) and had little effect on body weight (Figure 2(c)).

As the damage of islet *β* cells has a strong connection with pancreatic inflammation, we further studied the effect of BefA on the inflammatory signaling pathway, and our results indicated that 50 *μ*g BefA significantly downregulated the expressions of both TLR-4 and p-NF*κ*B (*p* < 0.05) (Figure 2(d)), and both 10 *μ*g BefA and 50 *μ*g BefA had effectively reduced the productions of IL-1*β* and TNF-*α* at a transcriptional level (*p* < 0.05) (Figures 2(e) and 2(f)). The H&E staining of mice pancreas further confirmed that the BefA protein could obviously increase the number of islet *β* cells in a concentration-dependent manner (Figure 2(g)).

### 3.3. BefA Protein Could Reduce Pancreas Injury and Restore Intestinal Microbiota to Normal Levels in T1DM Mice

**Figure 3:**
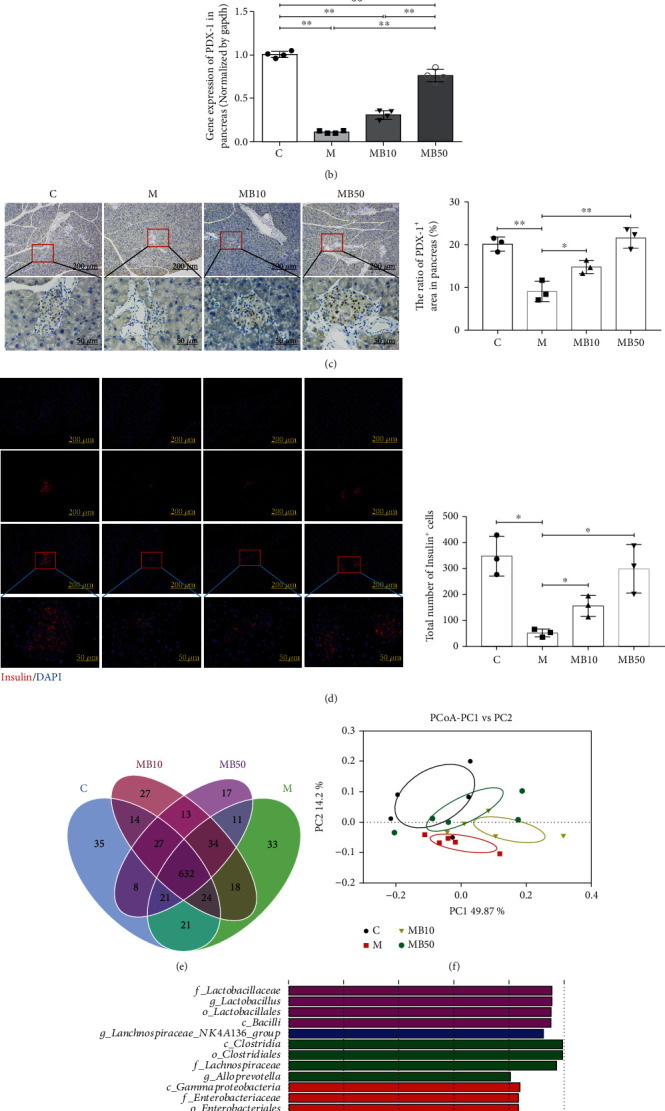
BefA protein could reduce pancreas injury and restore intestinal microbiota to normal levels in T1DM mice. (a) Western blotting results of the expression ratio of Bax/Bcl-2. (b) qPCR results of the transcription level of PDX-1. (c, d) The immunohistochemistry analysis of PDX-1 (×100 and ×400) and the immunofluorescence analysis of insulin (×100 and ×400) in the pancreas. The red area marks secreted insulin, which is used to label islet *β* cells. High-throughput sequencing results, including the total number of core operational taxonomic units (OTUs) shared and unique, in a Venn diagram (e), PcoA analysis of different group's samples (f), and LEfSe analysis for significantly different species (g) of T1DM mice faces among different groups. C group: wild-type C57BL/6 mice (*n* = 15); M group: T1DM model group treated with 0.9% physiological saline containing 0.01% gelatine administered intragastrically every other day for 14 times (*n* = 15); MB10 group: T1DM model group treated with 0.9% physiological saline containing 0.01% gelatine and 10 *μ*g BefA administered intragastrically every other day for 14 times (*n* = 15); and MB50 group: T1DM model group treated with 0.9% physiological saline containing 0.01% gelatine and 50 *μ*g BefA administered intragastrically every other day for 14 times (*n* = 15). Data are presented as mean ± SD. ^∗^*p* < 0.05, ^∗∗^*p* < 0.01.

To evaluate the effect of BefA on the pancreas of T1DM mice, some key proteins associated with apoptosis were tested. As shown in Figure 3(a), injection of STZ obviously induced apoptosis in the pancreas, and the use of both 10 *μ*g BefA and 50 *μ*g BefA significantly reduced cell necrosis in the pancreas by 48% and 66%, respectively, compared with the M group (*p* < 0.01). The BefA protein also significantly enhanced the expression of PDX-1 compared with the M group (*p* < 0.01) (Figures 3(b) and 3(c)) and increased the insulin secretion level in a concentration-dependent manner (*p* < 0.05) (Figure 3(d)).

In the analysis of the effect of BefA on intestinal microbiota by using high-throughput sequencing, the Venn diagram indicated that 632 OTUs were determined to be common OTUs among all groups, accounting for 80.82% (C group, 632/782), 79.60% (M group, 632/794), 80.10% (MB10 group, 632/789), and 82.83% (MB50 group, 632/763), respectively (Figure 3(e)). The PCoA result revealed a closer distance between the samples in the C and MB50 groups (Figure 3(f)), indicating that BefA has a positive effect on restoring the intestinal microbial composition to normal levels and is characterized by the high abundance of probiotic *Lactobacillus* at the family (f), genus (g), and order (o) levels when compared to the other three groups (Figure 3(g)).

### 3.4. BefA Protein Showed a Sound Protective Effect in the Pancreas of T2DM Mice

**Figure 4:**
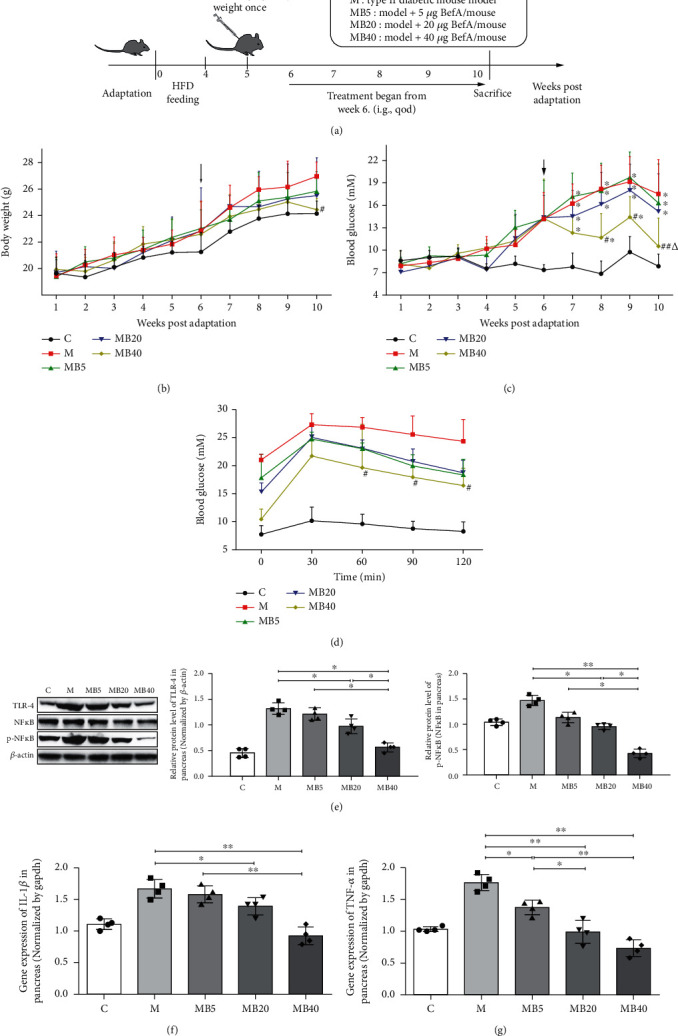
BefA protein showed sound protective effect on pancreas in T2DM mice. (a) Scheme of animal experiment (*n* = 15). (b) Body weight. (c) The blood glucose levels from mouse tail vein tested every week. (d) OGTT test performed on 10th week. (e) Western blotting results of the expression level of proinflammatory proteins including TLR-4 and the ratio of p-NF*κ*B/NF*κ*B in T2DM mouse pancreas. Gene expression levels of IL-1*β* (f) and TNF-*α* (g) in pancreas measured by q-PCR. The black arrows in (b) and (c) indicated that the BefA administration time started from the week 6. C group: mice fed with laboratory chow diet as the normal control group; M group: mice treated with 0.9% physiological saline containing 0.01% gelatine administered intragastrically every other day for 14 times (*n* = 15); MB5 group: mice treated with 0.9% physiological saline containing 0.01% gelatine and 5 *μ*g BefA (*n* = 15); MB20 group: mice treated with 0.9% physiological saline containing 0.01% gelatine and 20 *μ*g BefA (*n* = 15); MB40 group: mice treated with 0.9% physiological saline containing 0.01% gelatine and 40 *μ*g BefA (*n* = 15). Data are presented as mean ± SD. ^∗^*p* < 0.05, ^∗∗^*p* < 0.01.

To explore the anti-T2DM effect of BefA in mammals, a T2DM mice model was established and divided into five groups, including the C group (normal control group), M group (T2DM model group), MB5 group, MB10 group, and MB40 group (Figure 4(a)), and the effects of BefA on the body weight, blood glucose, and glucose tolerance of mice were tested. The results indicated that 40 *μ*g BefA could significantly reduce the weight gain symptoms of T2DM mice (*p* < 0.05) (Figure 4(b)), and both 20 *μ*g BefA and 40 *μ*g BefA could reduce the blood glucose level, of which a 41% reduction in the MB40 group was obtained at week 10 compared with that in the M group (*p* < 0.01) (Figure 4(c)). At week 10, oral glucose tolerance testing was performed, and 40 *μ*g BefA provided a significant improvement in glucose tolerance when compared with the M group 60 min after glucose injection (*p* < 0.05) (Figure 4(d)).

**Figure 5:**
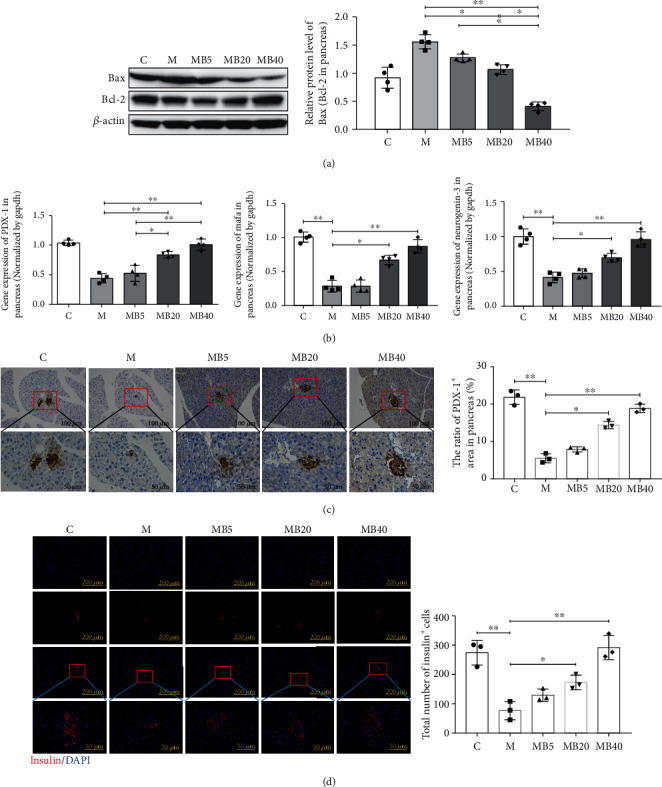
BefA protein showed a sound effect on increasing the number of islet *β* cells and insulin secretion in T2DM mice. (a) Western blotting results of the expression ratio of Bax/Bcl-2 in the pancreas. The gene transcription and protein expression levels of PDX-1 performed by qPCR (b) and immunohistochemistry analysis (c, ×200 and ×400). (d) Results of pancreatic immunofluorescence staining targeting insulin among different groups (×100 and ×400). C group: mice fed with laboratory chow diet as the normal control group; M group: mice treated with 0.9% physiological saline containing 0.01% gelatine administered intragastrically every other day for 14 times (*n* = 15); MB5 group: mice treated with 0.9% physiological saline containing 0.01% gelatine and 5 *μ*g BefA (*n* = 15); MB20 group: mice treated with 0.9% physiological saline containing 0.01% gelatine and 20 *μ*g BefA (*n* = 15); and MB40 group: mice treated with 0.9% physiological saline containing 0.01% gelatine and 40 *μ*g BefA (*n* = 15). Data are presented as mean ± SD. ^∗^*p* < 0.05, ^∗∗^*p* < 0.01.

Due to the important role of chronic systemic inflammation in the occurrence and development of T2DM, several key inflammatory proteins were detected. As shown in Figures 4(e)–4(g), key inflammatory factors, including TLR-4, p-NF*κ*B/NF*κ*B, IL-1*β*, and TNF-*α*, in the pancreas were significantly downregulated by the BefA protein in a concentration-dependent manner. Compared with the M group, 40 *μ*g BefA significantly reduced the Bax/Bcl-2 ratio from 1.55 to 0.43 (*p* < 0.01) (Figure 5(a)) and increased the PDX-1 transcription level from 0.43 to 1.01 (*p* < 0.01) (Figure 5(b)), which is consistent with the PDX-1 expression level in Figure 5(c). Mafa and neurogenin-3 also show concentration-dependent upward expression trends in Figure 5(b). In addition, immunofluorescence testing targeting insulin showed that the BefA protein had a sound effect on promoting insulin secretion levels in a concentration-dependent manner (Figure 5(d)).

### 3.5. BefA Protein Showed a Sound Effect on Regulating Liver Lipid Metabolism in T2DM Mice

**Figure 6:**
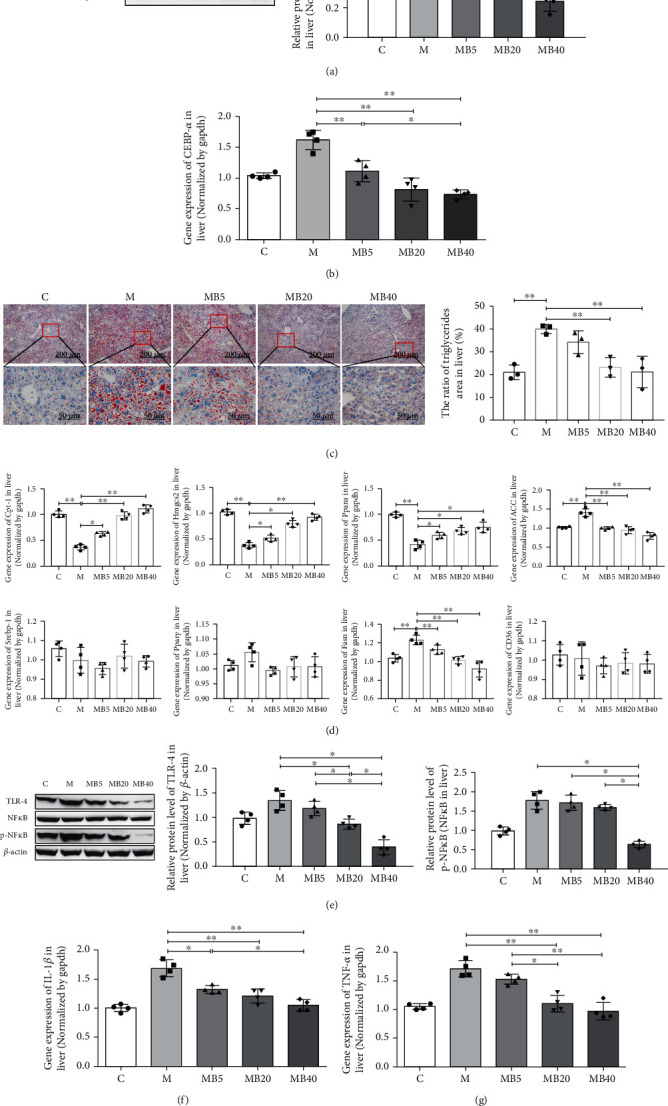
BefA protein showed a sound effect on regulating liver lipid metabolism in T2DM mice. (a, b) The change of the expression and transcription levels of the CEBP-*α* gene measured by Western blotting and qPCR. (c) The microscopic structure of the liver tissue by Oil Red O staining (×100 and ×400). (d) qPCR results of lipid metabolism-related factors, including Cpt-1, Hmgcs2, Ppar*α*, ACC, Srebp-1, Ppar*γ*, Fasn, and CD36 in the liver (*n* = 4). (e) Western blotting results of the expression levels of proinflammatory proteins, including TLR-4, and the ratio of p-NF*κ*B/NF*κ*B in the livers of T2DM mice. Gene expression levels of IL-1*β* (f) and TNF-*α* (g) in livers measured by qPCR. C group: mice fed with laboratory chow diet as the normal control group; M group: mice treated with 0.9% physiological saline containing 0.01% gelatine administered intragastrically every other day for 14 times (*n* = 15); MB5 group: mice treated with 0.9% physiological saline containing 0.01% gelatine and 5 *μ*g BefA (*n* = 15); MB20 group: mice treated with 0.9% physiological saline containing 0.01% gelatine and 20 *μ*g BefA (*n* = 15); and MB40 group: mice treated with 0.9% physiological saline containing 0.01% gelatine and 40 *μ*g BefA (*n* = 15). Data are presented as mean ± SD. ^∗^*p* < 0.05, ^∗∗^*p* < 0.01.

Because of the high occurrence of fatty liver and liver inflammation in T2DM mice, we evaluated the effect of BefA on liver function. The results indicated that BefA significantly downregulated the expression level of CEBP-*α* (a protein promoting adipocyte differentiation and acceleration off at accumulation) from 0.64 in the M group to 0.25 in the MB40 group (*p* < 0.01) (Figure 6(a)) and significantly reduced the CEBP-*α* expression at the transcriptional level in a concentration-dependent manner (Figure 6(b)). Moreover, the liver Oil Red O staining results indicated that a better fat-reducing effect was observed in groups with a higher concentration of BefA (*p* < 0.01) (Figure 6(c)). In Figure 6(d), we find that BefA increases the mRNA expression of genes involved in the regulation of the *β*-oxidation of fatty acids, including carnitine palmitoyltransferase 1 (Cpt-1), 3-hydroxy-3-methylglutaryl-CoA synthase 2 (Hmgcs2), and peroxisome proliferator-activated receptor *α* (Ppar*α*), and inhibits the mRNA expression of lipogenesis-associated genes, including acetyl-CoA carboxylase (ACC) and fatty acid synthase (Fasn) in a concentration-dependent manner. In addition, the BefA protein also significantly downregulated the levels of key inflammatory proteins (TLR-4, p-NF*κ*B/NF*κ*B, IL-1*β*, and TNF-*α*) (*p* < 0.05) (Figures 6(e)–6(g)).

## 4. Discussion

DM is a chronic systemic metabolic disease caused by long-term genetic and environmental factors [35].Although the pathological study of DM concerning genetics, immunology, and endocrinology has been developed in recent years, preventive and therapeutic methods still remain limited, with drug resistance, side effects, and painful administration method, as well as high economic burden, causing negative influences on the treatment effects and quality of life of patients [11, 36, 37]. Therefore, it is of great importance to develop new oral DM drugs with minor side effects and low economic cost.

Although the causes and pathological features of T1DM and T2DM are different, they also have common features, such as irreversible damage to islet *β* cells [17, 18]. Therefore, the drug can delay the occurrence of both T1DM and T2DM directly from the origin if it can promote the proliferation of islet *β* cells. Previous study has indicated that BefA could promote the proliferation of juvenile islet *β* cells in zebrafish, and BefA homologues have been confirmed to exist in the human intestine that exert the same function [19], which shows the potential antidiabetes effect of BefA in mammalian DM.

Firstly, we generated the BL21-pet 28C-BefA strain to produce BefA and verified the increasing effect of BefA on the number of mammalian islet *β* cells for the first time using GF mice (Figure 1). Then, the T1DM mice model induced by STZ was established. STZ could increase reactive oxygen species (ROS) to accelerate islet *β* cell DNA damage and directly destroy islet *β* cells, sharing typical human T1DM symptoms [38]. As *β* cell damage mainly occurs in the setting of islet cell inflammation and the TLR-4/NF*κ*B inflammation pathway is prodiabetic, whose high expression will trigger the release of inflammatory factors IL-1*β* and TNF-*α*, proinflammatory factors will inhibit the function of islet *β* cells and enhance cytotoxicity, which eventually leads to irreversible damage in a large number of islet *β* cells [39]. Consistent with results in zebrafish, BefA significantly reduced the blood glucose level, exerted a protective function on islet *β* cell morphology and cell density, and downregulated the expression levels of TLR-4 and p-NF*κ*B/NF*κ*B and the secretion levels of IL-1*β* and TNF-*α* in a concentration-dependent manner (Figure 2). T1DM is characterized by abnormal islet *β* cell apoptosis; therefore, we tested the expressions of Bax/Bcl-2 and PDX-1 in pancreatic tissue. Bax and Bcl-2 are classified as members of the Bcl-2 family, among which Bax is upregulated in apoptosis, Bcl-2 is an important antiapoptotic protein, and the Bax/Bcl-2 ratio is often used to evaluate their combined effect [40]. PDX-1, also called insulin promotor 1, is considered an irreplaceable transcriptional factor in the differentiation and proliferation of islet *β* cells [41]. The results indicated that BefA could recover the number of islet *β* cells and islet function of T1DM mice via reducing the Bax/Bcl-2 ratio, increasing the expression of PDX-1 and promoting insulin secretion (Figure 3). The insulin secretion did not be direct examined, and this should be a limitation of this study. Nevertheless, the examination of insulin using immunofluorescence may provide some clues to explain this issue.

More and more studies have indicated that intestinal microbiota are closely related to the development of DM [42, 43]; so, we conducted a high-throughput sequencing analysis of intestinal microbiota in mice with T1DM. The results showed that BefA could restore the disturbed microbial composition in the M group to normal levels and markedly increased the abundance of probiotic lactic acid bacteria (LAB) in the MB50 group (Figure 3). LAB, such as *Lactobacillus* and *Bifidobacterium*, can inhibit pathogenic bacteria, protecting the barrier function of the intestinal wall as well as the health of the body [44, 45]. Moreover, LAB can decrease the blood glucose level by directly decomposing glucose and reducing the expression of the glucose transporter (GLUT) protein and inhibiting glucose absorption in the intestinal wall [22].

In T2DM mice, BefA also obviously reduced the blood glucose level and relieve weight gain symptoms in a concentration-dependent manner (Figure 4). Notably, insulin resistance, which is an important pathological indicator for evaluating glucose metabolism ability and insulin sensitivity in T2DM individuals [46], was significantly improved in the MB40 group, indicating that the BefA protein played a positive role in regulating blood glucose metabolism. Similar to the results in the T1DM mice model, oral administration of BefA reduced pancreatic inflammation by downregulating the expressions of TLR-4, p-NF*κ*B/NF*κ*B, IL-1*β*, and TNF-*α*, as well as promote the proliferation of islet *β* cells by lowering cell apoptosis (Bax/Bcl-2) and increasing the expression of PDX-1 (Figures 4 and 5). In addition, the results suggest that BefA can promote the *β*-oxidation of fatty acids by upregulating Cpt-1, Hmgcs2, and Ppar*α* and inhibit lipogenesis by downregulating the expressions of CEBP-*α*, ACC, and Fasn, as well as inhibit the synthesis of triglycerides, leading to improvements in both fatty liver and metabolic dysfunction in a concentration-dependent manner (Figure 6). Patients with T2DM can develop various complications, including fatty liver and liver inflammation, and dysfunction of hepatic lipid metabolism could be improved by activation of Cpt-1, Hmgcs2, and Ppar*α* to enhance hepatic oxidation and the metabolism of adipose tissue [47–49]. CEBP-*α*, ACC, and Fasn play important roles in initiating liver fat formation and lead to fatty liver [50–53].Another limitation of this study is the lack of lipid metabolism, we only discuss some indexes connected with diabetes for the liver, and so further studies on lipid metabolism will be needed to explain how BefA improve lipid metabolism.

## 5. Conclusion

**Figure 7:**
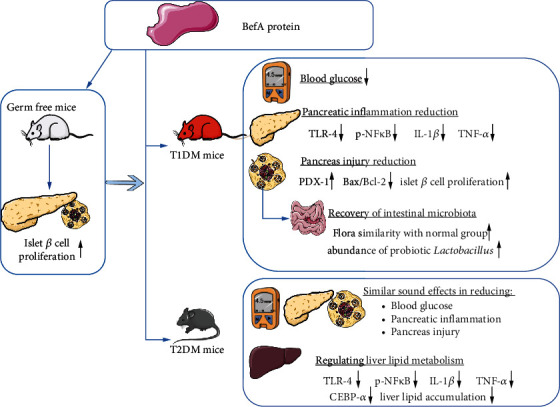
Mechanism diagram of BefA protein on diabetes.

In summary, the present study reveals the antidiabetes effects of BefA in mammals for the first time, via increasing the number of islet *β* cells, reducing the inflammatory reaction and apoptosis, improving liver lipid metabolism, and restoring microbial diversity to normal levels, which provides a new strategy for an oral drug for DM via inhibiting the progression of islet *β* cell destruction in DM(Figure 7). However, a deeper understanding of the underlying mechanisms and the use of an engineered strain to replace the continuous administration of BefA is necessary for further study [54, 55].

## Figures and Tables

**Table 1:** Primers for amplifying different genes via qPCR.

Target primers	Sequence (5′ to 3′)
IL-1*β*	F: GTGTCTTT CCCGTGGACCTTC
	R: TCATCTCGGAGCCTGTAGTGC

TNF-*α*	F: GTGGAACTGGCAGAAGAGGCA
	R: AGAGGGAGGCCATTTGGGAAC

PDX-1	F: CGCGGTTCTATTTTGTTGGT
	R: AGTCGGCATCGTTTATGGTC

CEBP-*α*	F: TAGGTTTCTGGGCTTTGTGG
	R: AGCCGTTAGTGAAGAGTCTCAGTTT

GAPDH	F: CTCGTGGAGTCTACTGGTGT
	R: GTCATCATACTTGGCAGGTT

Mafa	F: GCTTCAGCAAGGAGGAGGTCAT
	R: TCTCGCTCTCCAGAATGTGCCG

Neurogenin-3	F: TCCGAAGCAGAAGTGGGTGACT
	R: CGGCTTCTTCGCTGTTTGCTGA

Cpt-1	F: GGCATAAACGCAGAGCATTCCTG
	R: CAGTGTCCATCCTCTGAGTAGC

Hmgcs2	F: GCTCGTCTACAGAAACTCCACG
	R: GCTTCAGCAGTGCTTTCTCCGT

Ppar*α*	F: ACCACTACGGAGTTCACGCATG
	R: GAATCTTGCAGCTCCGATCACAC

ACC	F: GTTCTGTTGGACAACGCCTTCAC
	R: GGAGTCACAGAAGCAGCCCATT

Srebp-1	F: CGACTACATCCGCTTCTTGCAG
	R: CCTCCATAGACACATCTGTGCC

Ppar*γ*	F: GTACTGTCGGTTTCAGAAGTGCC
	R: ATCTCCGCCAACAGCTTCTCCT

Fasn	F: CACAGTGCTCAAAGGACATGCC
	R: CACCAGGTGTAGTGCCTTCCTC

CD36	F: GGACATTGAGATTCTTTTCCTCTG
	R: GCAAAGGCATTGGCTGGAAGAAC

## Data Availability

The authors confirm that the data supporting the findings of this study are available within the article and its supplementary materials. Raw sequences have been deposited in the GenBank under accession number PRJNA637680.

## Supplementary Materials

Supplementary Materials Figure S1: experimental replications for Western blotting experiments. (a, b) Experimental replications for Figures 2(d) and 3(a), carried out by T1DM mice pancreas. (c, d) Experimental replications for Figures 4(e) and 5(a), carried out by T2DM mice pancreas. (e, f) Experimental replications for Figures 6(a) and 6(e), carried out by T2DM mice liver.

## References

[1] Association A. D. (2012). Diagnosis and classification of diabetes mellitus. *Recenti Progressi in Medicina*.

[2] Wang T., Lu J., Shi L. (2020). Association of insulin resistance and *β*-cell dysfunction with incident diabetes among adults in China: a nationwide, population-based, prospective cohort study. *The lancet Diabetes & endocrinology*.

[3] Sattar N. (2013). Revisiting the links between glycaemia, diabetes and cardiovascular disease. *Diabetologia*.

[4] Kumar P., Sakwariya A., Sultania A. R., Dabas R. (2017). Hypertriglyceridemia-induced acute pancreatitis with diabetic ketoacidosis: a rare presentation of type 1 diabetes mellitus. *Journal of Laboratory Physicians*.

[5] Cho N. H., Shaw J. E., Karuranga S. (2018). IDF diabetes atlas: global estimates of diabetes prevalence for 2017 and projections for 2045. *Diabetes Research and Clinical Practice*.

[6] Zimmet P. Z., Magliano D. J., Herman W. H., Shaw J. E. (2014). Diabetes: a 21st century challenge. *The Lancet Diabetes & Endocrinology*.

[7] Iwasaki M., Sato M., Yoshihara A., Miyazaki H. (2016). Effects of periodontal diseases on diabetes-related medical Expenditure. *Current Oral Health Reports*.

[8] Jahromi M. M., Eisenbarth G. S. (2007). Cellular and molecular pathogenesis of type 1A diabetes. *Cellular and Molecular Life Sciences*.

[9] Weir G. C., Bonner-Weir S. (2013). Islet *β* cell mass in diabetes and how it relates to function, birth, and death. *Annals of the New York Academy of Sciences*.

[10] Amiel S. A., Rela M. (2005). Live organ-donation for islet transplantation. *Lancet*.

[11] Song S., Roy S. (2016). Progress and challenges in macroencapsulation approaches for type 1 diabetes (T1D) treatment: cells, biomaterials, and devices. *Biotechnology and Bioengineering*.

[12] Im G. B., Bhang S. H. (2018). Recent research trend in cell and drug delivery system for type 1 diabetes treatment. *Journal of Pharmaceutical Investigation*.

[13] Song W. Q., Fu D. Z., Cheng Y., Liu Y. F. (2015). Influence of adenosine on preservation of porcine pancreas in islet transplantation. *Genetics & Molecular Research*.

[14] Inagaki N., Araki E., Oura T., Matsui A., Takeuchi M., Tanizawa Y. (2016). The combination of dulaglutide and biguanide reduced bodyweight in Japanese patients with type 2 diabetes. *Diabetes, Obesity & Metabolism*.

[15] Hsu C. C., Wahlqvist M. L., Lee M. S., Tsai H. N. (2011). Incidence of dementia is increased in type 2 diabetes and reduced by the use of sulfonylureas and metformin. *Journal of Alzheimer's Disease*.

[16] de Wit H. M., te Groen M., Rovers M. M., Tack C. J. (2016). The placebo response of injectable GLP-1 receptor agonistsvs. oral DPP-4 inhibitors and SGLT-2 inhibitors: a systematic review and meta-analysis. *British Journal of Clinical Pharmacology*.

[17] Elizabeth M. M., Alarcon-Aguilar B. S. P., Clara O. C., Escobar-Villanueva B. S. P., Carmen M. (2017). Pancreatic *β*-cells and type 2 diabetes development. *Current Diabetes Reviews*.

[18] Mirmira R. G., Sims E. K., Syed F., Evans-Molina C. (2016). Biomarkers of *β*-cell stress and death in type 1 diabetes. *Current Diabetes Reports*.

[19] Hill J. H., Franzosa E. A., Huttenhower C., Guillemin K. (2016). A conserved bacterial protein induces pancreatic beta cell expansion during zebrafish development. *eLife*.

[20] Tai N., Wong F. S., Wen L. (2016). The role of the innate immune system in destruction of pancreatic beta cells in NOD mice and humans with type I diabetes. *Journal of Autoimmunity*.

[21] Oliver-Krasinski J. M., Kasner M. T., Yang J. (2009). The diabetes gene Pdx1 regulates the transcriptional network of pancreatic endocrine progenitor cells in mice. *Journal of Clinical Investigation*.

[22] Wang L., Shang Q., Guo W. (2020). Evaluation of the hypoglycemic effect of probiotics via directly consuming glucose in intestines of STZ-induced diabetic mice and glucose water-induced diabetic mice. *Journal of Functional Foods*.

[23] Zhu Y., Liu Q., Zhou Z., Ikeda Y. (2017). PDX1, Neurogenin-3, and MAFA: critical transcription regulators for beta cell development and regeneration. *Therapy*.

[24] Badawi A. (2011). Vitamins D, C, and E in the prevention of type 2 diabetes mellitus: modulation of inflammation and oxidative stress. *Biologics: Targets & Therapy*.

[25] Wen L., Ley R. E., Volchkov P. Y. (2008). Innate immunity and intestinal microbiota in the development of type 1 diabetes. *Nature*.

[26] Jiao P., Ma J., Feng B. (2011). FFA-induced adipocyte inflammation and insulin resistance: involvement of ER stress and IKK*β* pathways. *Obesity*.

[27] de Freitas Moreira M., do Vale Canabrava N., Lira S. M. (2015). Experimental model of type 2 diabetes induced by fat diet consumption and low dose of streptozotocin in C57BL/6J mice. *Diabetology & Metabolic Syndrome*.

[28] Ghiasi R., Ghadiri Soufi F., Somi M. . (2015). Swim training improves HOMA-IR in type 2 diabetes induced by high fat diet and low dose of streptozotocin in male Rats. *Pharmaceutical Bulletin*.

[29] Baribault H. (2016). Mouse models of type 2 diabetes mellitus in drug discovery. *Mouse Models for Drug Discovery*.

[30] Mariño E., Richards J. L., McLeod K. H. (2017). Gut microbial metabolites limit the frequency of autoimmune T cells and protect against type 1 diabetes. *Nature Immunology*.

[31] Ji Y., Sun S., Shrestha N. (2019). Toll-like receptors TLR2 and TLR4 block the replication of pancreatic *β* cells in diet-induced obesity. *Nature Immunology*.

[32] Chen T., Zhao X., Ren Y. (2019). Triptolide modulates tumour-colonisation and anti-tumour effect of attenuated salmonella encoding DNase I. *Applied Microbiology and Biotechnology*.

[33] Geller L. T., Barzily-Rokni M., Danino T. (2017). Potential role of intratumor bacteria in mediating tumor resistance to the chemotherapeutic drug gemcitabine. *Science*.

[34] Chen T., Tian P., Huang Z. (2018). Engineered commensal bacteria prevent systemic inflammation-induced memory impairment and amyloidogenesis via producing GLP-1. *Applied Microbiology and Biotechnology*.

[35] Saeedi P., Petersohn I., Salpea P. (2019). Global and regional diabetes prevalence estimates for 2019 and projections for 2030 and 2045: Results from the International Diabetes Federation Diabetes Atlas, 9^th^ edition. *Diabetes Research and Clinical Practice*.

[36] Ewald N. H., Giessen P. D. (2013). Diagnosis and treatment of diabetes mellitus in chronic pancreatitis. *World Journal of Gastroenterology*.

[37] Kheirandish M., Mahboobi H., Yazdanparast M., Kamal M. (2017). Challenges related to glycemic control in type 2 diabetes mellitus patients. *Current Drug Metabolism*.

[38] Radenković M., Stojanović M., Prostran M. (2016). Experimental diabetes induced by alloxan and streptozotocin: the current state of the art. *Journal of Pharmacology and Toxicology*.

[39] Yang J., Sun Y., Xu F. (2018). Involvement of estrogen receptors in silibinin protection of pancreatic *β*-cells from TNF*α*- or IL-1*β*-induced cytotoxicity. *Biomedicine & Pharmacotherapy*.

[40] Tomita T. (2017). Apoptosis of pancreatic *β* cells in type 1 diabetes. *Bosnian Journal of Basic Medical Sciences*.

[41] Holland A. M., Góñez L. J., Naselli G., MacDonald R. J., Harrison L. C. (2005). Conditional expression demonstrates the role of the homeodomain transcription factor Pdx1 in Maintenance and regeneration of *β*-Cells in the adult pancreas. *Diabetes*.

[42] Paschou S. A., Papadopoulou-Marketou N., Chrousos G. P., Kanaka-Gantenbein C. (2018). On type 1 diabetes mellitus pathogenesis. *Endocrine Connections*.

[43] Hu C., Wong F. S., Wen L. (2015). Type 1 diabetes and gut microbiota: friend or foe?. *Pharmacological Research*.

[44] Cálix-Lara T. F., Rajendran M., Talcott S. T. (2014). Inhibition of *Escherichia coli* O157:H7 and *Salmonella enterica* on spinach and identification of antimicrobial substances produced by a commercial Lactic Acid Bacteria food safety intervention. *Food Microbiology*.

[45] Li X., Wang N., Yin B. (2016). Lactobacillus plantarum X1 with *α*-glucosidase inhibitory activity ameliorates type 2 diabetes in mice. *RSC Advances*.

[46] Saini V. (2010). Molecular mechanisms of insulin resistance in type 2 diabetes mellitus. *World Journal of Diabetes*.

[47] Montagner A., Polizzi A., Fouché E. (2016). Liver PPAR*α* is crucial for whole-body fatty acid homeostasis and is protective against NAFLD. *Gut*.

[48] Lundsgaard A. M., Fritzen A. M., Kiens B. (2018). Molecular regulation of fatty acid oxidation in skeletal muscle during aerobic exercise. *Trends in Endocrinology and Metabolism*.

[49] Vilà-Brau A., de Sousa-Coelho A. L., Mayordomo C., Haro D., Marrero P. F. (2011). Human HMGCS2 Regulates Mitochondrial Fatty Acid Oxidation and *FGF21* Expression in HepG2 Cell Line∗. *The Journal of Biological Chemistry*.

[50] Hadrich F., Sayadi S. (2018). Apigetrin inhibits adipogenesis in 3T3-L1 cells by downregulating PPAR*γ* and CEBP-*α*. *Lipids in Health and Disease*.

[51] Luo L., Fang K., Dan X., Gu M. (2019). Crocin ameliorates hepatic steatosis through activation of AMPK signaling in db/db mice. *Lipids in Health and Disease*.

[52] Horton J. D., Bashmakov Y., Shimomura I., Shimano H. (1998). Regulation of sterol regulatory element binding proteins in livers of fasted and refed mice. *Proceedings of the National Academy of Sciences of the United States of America*.

[53] Jensen-Urstad A. P., Semenkovich C. F. (2012). Fatty acid synthase and liver triglyceride metabolism: housekeeper or messenger?. *Biochimica et Biophysica Acta*.

[54] Anonymous (2006). Transgenic bacteria can be used to deliver IL-10 to gut mucosa. *Gastroenterology & Hepatology*.

[55] Martín R., Martín R., Chain F. (2014). Effects in the use of a genetically engineered strain ofLactococcus lactisdelivering in situ IL-10 as a therapy to treat low-grade colon inflammation. *Human Vaccines & Immunotherapeutics*.

[56] Wang H., Wei J., Hu H. (2021). *Oral administration of bacterial beta cell expansion factor A (BefA) alleviates diabetes in mice with type 1 and type 2 diabetes*.

