# Supplementary figures and images for: Oral Administration of Bacterial β Cell Expansion Factor A (BefA) Alleviates Diabetes in Mice with Type 1 and Type 2 Diabetes
Source: Oxid Med Cell Longev. 2022 Feb 10;2022:9206039. doi: 10.1155/2022/9206039 (PMC8853770; doi:10.1155/2022/9206039)

**a**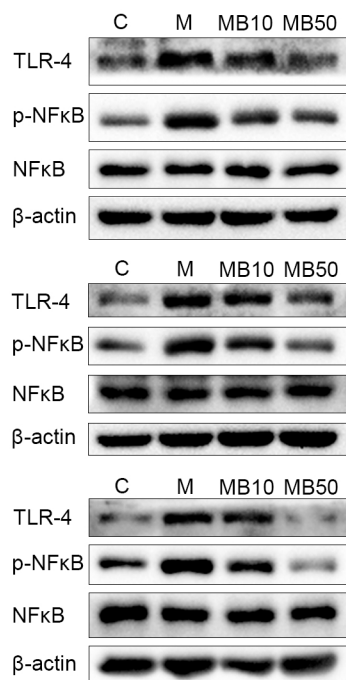**b**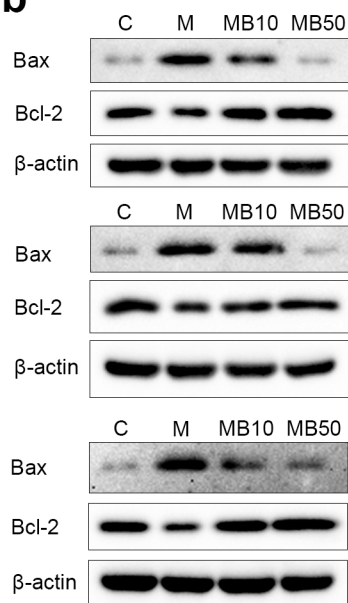**c**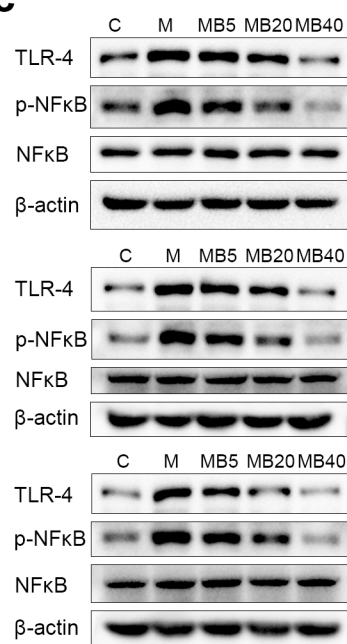**d**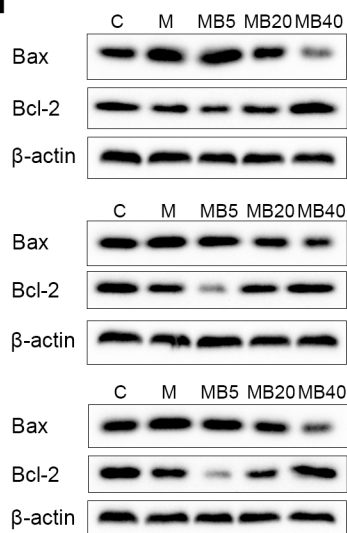**e**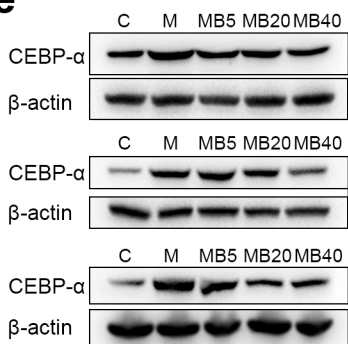**f**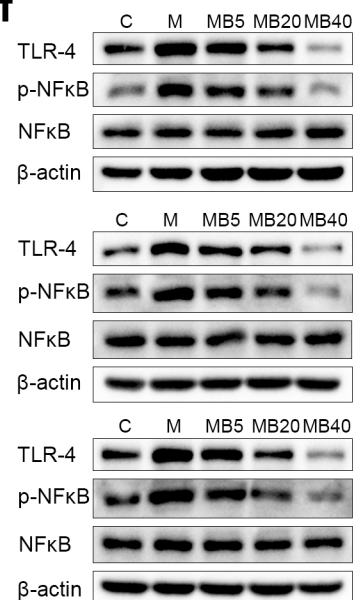

Supplement: Supplementary Materials — Figure S1: experimental replications for Western blotting experiments. (a, b) Experimental replications for Figures 2(d) and 3(a), carried out by T1DM mice pancreas. (c, d) Experimental replications for Figures 4(e) and 5(a), carried out by T2DM mice pancreas. (e, f) Experimental replications for Figures 6(a) and 6(e), carried out by T2DM mice liver. [file 9206039.f1.pdf]
